# Supplementary material for: Adaptative Divergence of Cryptococcus neoformans: Phenetic and Metabolomic Profiles Reveal Distinct Pathways of Virulence and Resistance in Clinical vs. Environmental Isolates
Source: J Fungi (Basel). 2025 Mar 12;11(3):215. doi: 10.3390/jof11030215 (PMC11943092; doi:10.3390/jof11030215)
Supplement: Supplementary file 1 [file jof-11-00215-s001.zip › jof-3375313-supplementary.pdf]

Table S1. Metabolites associated with glycolytic enzymes in *C. neoformans* strains: comparative abundance in clinical and environmental isolates.

**Metabolites associated with glycolytic enzymes in higher abundance in clinical strains**			
Code	Description for *C. neoformans*	Matches - "Human"	Pathway product
HMDB0000588	OXL05196.1 alpha-glucosidase	NabaatprBAT	Sodium
HMDB0000192			L-Cystine
HMDB0250712			Cystine
HMDB0000464	OXL05196.1 alpha-glucosidase	4F2 cell-surface antigen heavy chain	Calcium
HMDB0000588			Sodium
HMDB0000181			DOPA
HMDB0000248			Thyroxine
HMDB0000574			L-Cysteine
HMDB0000687			Leucine
HMDB0000517			L-Arginine
HMDB0001254	OXH03273.1 N-acetylglucosamine-6-phosphate deacetylase	PN-a-6-pd	Glucosamine 6-phosphate
HMDB0001062			N-Acetyl-D-Glucosamine 6-Phosphate
HMDB0000042			Acetic acid
HMDB0002111			Water
HMDB0000588	OXH09771.1 alpha-glucosidase	NabaatprBAT	Sodium
HMDB0000192			L-Cystine
HMDB0250712			Cystine
HMDB0000464	OXH09771.1 alpha-glucosidase	4F2 cell-surface antigen heavy chain	Calcium
HMDB0000588			Sodium
HMDB0000181			DOPA
HMDB0000248			Thyroxine
HMDB0000574			L-Cysteine
HMDB0000687			Leucine
HMDB0000517			L-Arginine
**Metabolites associated with glycolytic enzymes in higher abundance in environmental strains**			
Code	Description for *C. neoformans*	Matches - "H*uman*"	Pathway product
HMDB0001550	UOH85264.1 S-formylglutathione hydrolase	S-formylglutathione hydrolase	S-Formylglutathione
HMDB0000125			Glutathione
HMDB0002111			Water
HMDB0000142			Formic acid
HMDB0032989			4-Methylumbelliferyl acetate
HMDB0059622			4-Methylumbelliferone
HMDB0000042			Acetic acid

NabaatprBAT = Neutral and basic amino acid transport protein rBAT; PN-a-6-pd = Putative N-acetylglucosamine-6-phosphate deacetylase
